# Behind the silence of harmony: risk factors for physical and sexual violence among women in rural Indonesia

**DOI:** 10.1186/1472-6874-11-52

**Published:** 2011-11-23

**Authors:** Elli N Hayati, Ulf Högberg, Mohammad Hakimi, Mary C Ellsberg, Maria Emmelin

**Affiliations:** 1 Rifka Annisa Women's Crisis Center, Yogyakarta, Indonesia; 2 Center for Health and Nutrition Research Laboratory, Gadjah Mada University, Yogyakarta, Indonesia; 3 Faculty of Psychology, Ahmad Dahlan University, Yogyakarta, Indonesia; 4 Epidemiology and Global Health, Department of Public Health and Clinical Medicine, Umeå University, Umeå Sweden; 5 Women's and Childrens' Health, Uppsala University, Uppsala Sweden; (Akademiska Sjukhuset, Ing. 96 nbv, SE-751 85 Uppsala, Sweden; 6 ICRW (International Center for Research on Women), Washington, D.C., USA; (1120 20th St NW, Suite 500 North, Washington, D.C. 20036, USA; 7 Social Medicine and Global Health, Department of Clinical Sciences, Lund University, Lund, Sweden. (CRC, Entrance 72, 205 02 Malmö, Sweden

## Abstract

**Background:**

Indonesia has the fourth largest population in the world. Few studies have identified the risk factors of Indonesian women for domestic violence. Such research will be useful for the development of prevention programs aiming at reducing domestic violence. Our study examines associations between physical and sexual violence among rural Javanese Indonesian women and sociodemographic factors, husband's psychosocial and behavioral characteristics and attitudes toward violence and gender roles.

**Methods:**

A cohort of pregnant women within the Demographic Surveillance Site (DSS) in Purworejo district, Central Java, Indonesia, was enrolled in a longitudinal study between 1996 and 1998. In the following year (1999), a cross-sectional domestic violence household survey was conducted with 765 consenting women from that cohort. Female field workers, trained using the WHO Multi-Country study instrument on domestic violence, conducted interviews. Crude and adjusted odds ratios at 95% CI were applied for analysis.

**Results:**

Lifetime exposure to sexual and physical violence was 22% and 11%. Sexual violence was associated with husbands' demographic characteristics (less than 35 years and educated less than 9 years) and women's economic independence. Exposure to physical violence among a small group of women (2-6%) was strongly associated with husbands' personal characteristics; being unfaithful, using alcohol, fighting with other men and having witnessed domestic violence as a child. The attitudes and norms expressed by the women confirm that unequal gender relationships are more common among women living in the highlands and being married to poorly educated men. Slightly more than half of the women (59%) considered it justifiable to refuse coercive sex. This attitude was also more common among financially independent women (71%), who also had a higher risk of exposure to sexual violence.

**Conclusions:**

Women who did not support the right of women to refuse sex were more likely to experience physical violence, while those who justified hitting for some reasons were more likely to experience sexual violence. Our study suggests that Javanese women live in a high degree of gender-based subordination within marriage relationships, maintained and reinforced through physical and sexual violence. Our findings indicate that women's risk of physical and sexual violence is related to traditional gender norms.

## Background

Although violence against women has been increasingly recognized as a significant international human rights and public health problem, most of the research on the subject conducted prior to 2000 did not use standardized methods and tools allowing findings to be compared across settings. The WHO-Multi-Country Study on Women's Health and Domestic Violence against Women represented a milestone for the field, because it used a common research design and a standardized questionnaire validated for various cultural settings. The first stage of the study involved over 24,000 women in 10 countries: Bangladesh, Brazil, Ethiopia, Japan, Namibia, Peru, Samoa, Serbia and Montenegro, Thailand, and Tanzania. The study addressed the prevalence of physical, sexual and emotional violence experienced by women aged 15-49, sociodemographic risk factors, health outcomes, women's responses to violence, as well as gender-related norms. The WHO study showed that lifetime and current prevalence of physical violence against women by an intimate partner ranged from 12.9-48.7% and 3.1-29%, respectively in the participating countries, while the corresponding range for lifetime and current prevalence of sexual violence was 6.3-49.7% and 1.1-29.0% [1].

A cross-sectional survey of violence against women based on the WHO study methodology was conducted in rural Indonesia. The report on the study's findings entitled "Silence for the Sake of Harmony" revealed that the lifetime and current prevalence of physical violence among reproductive age women by an intimate partner were 11% and 2%, and the lifetime and current prevalence of sexual violence were 22% and 12% [2]. The Indonesian figures for physical violence were thus lower than in the WHO sites, although the ones for sexual violence fell in the middle range. Similar to the other south Asian sites of the WHO study (Thailand and Bangladesh), the prevalence of sexual violence was higher than physical violence [1].

Indonesia has the fourth largest population in the world. It is rich in cultural differences, is spread across several islands and 88% of its population is Muslim. The cross-sectional study's report [2] was used to advocate for policy changes in Indonesia, and resulted in the endorsement of the Domestic Violence Act in 2004. However, a national study performed by Rifka Annisa Women's Crisis Center indicated a weak implementation of this Act [3]. Using the existing cross sectional data, our study examines the associations between women's domestic violence experience and sociodemographic factors, husband's psychosocial and behavioral characteristics, as well as women's attitudes toward gender roles and the use of violence within marriage.

## Methods

### Study Site and Sample

The study was linked to a Demographic Health Surveillance (DHS) site in Purworejo district, set up in 1992 by the Center for Health and Nutrition Research Laboratory (CHN-RL) and attached to the faculty of Medicine, Gadjah Mada University, Yogyakarta, Indonesia. Women of reproductive age (15-49 years), who were already enrolled in a longitudinal pregnancy study (1996-1998), were asked to participate in this cross-sectional domestic violence survey [2]. From a total of 846 women in that cohort, 27 had moved out of the area, 5 had died and 49 refused. Finally 765 consented to be interviewed for the study.

### Data collection

The WHO multi-country questionnaire was translated into the Indonesian language and pretested to ensure that the content was culturally appropriate for the setting. The data were collected from December 1999 through February 2000 by 12 female field workers and two female supervisors who could speak the local language and had previous data collection experience. The research team specifically trained them to use the WHO questionnaire, using pilot interviews from outside the surveillance site. During the field work phase, questionnaires were checked daily by the field supervisors, and once approved, taken to Yogyakarta for data entry.

### Ethical and safety issues

The WHO guidelines on domestic violence research were applied throughout the study [4]. All field workers and supervisors underwent two weeks training to improve the quality of their data collection by including knowledge on gender-based violence, and skills on how to communicate empathetically and how to maintain privacy and security during interviews. The women field workers got weekly debriefings to discuss and reflect on their field experiences. All participants received a small brochure with a hotline number for counseling. Rifka Annisa Women's Crisis Center's counselors were assigned to Purworejo to run counseling services at the study participants' request.

### Data Management and Analysis

All datasets from the survey were maintained by the data management team from the Center for Health and Nutrition Research Laboratory (CHN-RL) at the Gadjah Mada University, Yogyakarta.

The WHO questionnaire consisted of sociodemographic variables, reproductive and health data, lifetime violence experiences, and gender related attitudes and norms. Below is a description of the variables analyzed in our study.

Experience of Violence referred to physical and sexual violence by a current or former intimate partner. A woman who reported having been slapped, hit by an object, pushed, dragged, kicked, or beaten by her husband was regarded as having experienced physical abuse. A woman who had been physically forced to have sex when she did not want to, or had sex because she was afraid of what her husband might do or had been forced to perform sexually degrading acts was regarded as having experienced sexual abuse.

Lifetime prevalence referred to a woman having experienced one or more of the acts described above at any time during her life. Current prevalence referred to experiencing any of the described acts within the 12 months prior to the interview.

The sociodemographic variables chosen for our analysis were women's and husband's age and education, number of children, residence (highland or lowland), and husband's type of work (agricultural or non-agricultural).

Husband's psychosocial and behavioral characteristics included having witnessed his mother being hit by his father, being willing to share his income with his wife, stealing his wife's money, being unfaithful, fighting with other men, and being drunk often.

Attitudes toward gender roles consisted of six statements, with which the women were asked to agree or disagree. The statements included "a good wife obeys her husband", "family problems should only be discussed with a family member", "a man should show who is the boss", "a wife is obliged to have sex with her husband", "a woman should be able to choose her own friends" and "others outside the family should intervene (in case of violence)".

Justification for a husband to hit his wife was based on women's endorsement of six possible reasons why a husband would be justified in hitting his wife: "she does not do the household chores well"; "she disobeys him"; "she asks him about girlfriends"; "he suspects that she is unfaithful"; or "she is unfaithful". The variables were recoded into three categories: "hitting a wife is never justified"; "hitting a wife is justified for one reason only"; and "hitting a wife is justified for two or more reasons".

Justification for a wife to refuse sex included four statements regarding possible scenarios in which a woman has the right to refuse sex: "if she does not want to"; "if he is drunk"; "if she is sick"; or "if he mistreats her". In the analysis, a composite variable was created with three values: "to refuse sex is justified for all four reasons", "to refuse sex is justified for up to three reasons", and "to refuse sex is justified for one or no reason at all."

Crude and adjusted odds ratios (AOR) with 95% confidence intervals (CI) were calculated using the Statistical Package for the Social Sciences 15.0 (SPSS Inc., Chicago, IL) for bivariate and multivariate logistic regression analysis. In the multivariate analysis only significant variables from the bivariate analysis were analyzed.

## Results

Lifetime exposure to physical and sexual violence was reported to be 11% and 22%. The associations between exposure to physical and sexual violence, sociodemographic factors, and husband's characteristics are presented in Table 1. Woman's age, education, parity, or husband's type of work were not associated with women's reports of ever having experienced physical or sexual violence. Marital age and education gaps between the woman and her partner were also not associated with women's exposure to violence.

**Table 1:** Woman's and husband's characteristics in relation to exposure to physical and sexual violence, crude and adjusted odds ratios (95% CI).

	Characteristic of woman and her husband	All women N = 765	Physical violence n = 84		Sexual violence n = 165	
			OR (CI 95%)	**AOR**^**1**^ (95% CI)	OR (CI 95%)	**AOR**^**2**^ (CI 95%)
**Woman's age**	> 35 years	216 (28%)	1		1	
	≤ 35 years	549 (72%)	0.92 (0.56-1.52)		0.89 (0.61-1.29)	

**Woman's education**	> 9 years	305 (40%)	1		1	
	≤ 9 years	460 (60%)	0.92 (0.58-1.46)		1.38 (0.96-1.98)	

**Woman's economic independency**	No own income	609 (80%)	1		1	
	Has own income	156 (20%)	0.69 (0.37-1.27)		1.71* (1.15-2.55)	1.65* (1.08-2.52)

**Children**	1	134 (18%)	1		1	
	≥ 2	631 (82%)	1.07 (0.59-1.97)		0.9 (0.58-1.41)	

**Geographical area**	Lowland	432 (57%)	1		1	
	Highland	330 (43%)	0.97 (0.62-1.54)		2.04* (1.44-2.9)	0.53 (0.37-0.76)

**Husband's age**	> 35 years	410 (54%)	1		1	
	≤ 35 years	355 (46%)	1.17 (0.74-1.84)		1.65* (1.17-2.34)	1.71* (1.19-2.47)

**Husband's education**	> 9 years	254 (33%)	1		1	
	≤ 9 years	511 (67%)	0.99 (0.62-1.62)		1.96* (1.31-2.93)	2.07* (1.36-3.17)

**Husband's type of work**	Non Agricultural	345 (45%)	1		1	
	Agricultural	420 (55%)	1.94 * (1.19-3.16)	1.47 (0.85-2.54)	0.8 (0.57-1.14)	

**Husband stole her money**	Never	750 (98%)	1		1	
	Yes, Once or more	15 (2%)	5.73* (1.99-16.5)	2.84 (0.64-12.6)	1.83 (0.62-5.44)	

**Husband share his income**	Yes	726 (95%)	1		1	
	No	39 (5%)	2.56* (1.19-5.68)	0.95 (0.33-2.71)	2.68* (1.38-5.20)	1.96 (0.96-4.00)

**Husband has other woman**	No	726 (95%)	1		1	
	Yes	39 (5%)	13.8* (6.97-27.39)	8.15* (3.64-18.3)	3.36* (1.74-6.47)	2.31* (1.09-4.88)

**Husband drinks alcohol**	No	722 (94%)	1		1	
	Yes	43 (6%)	9.95* (5.19-19.1)	5.39* (2.46-11.8)	2.52* (1.33-4.77)	1.81 (0.88-3.73)

**Husband witnessed his mother being hit as a child**	No	749 (98%)	1		1	
	Yes	16 (2%)	15.2* (5.36-42.39)	11.6* (3.56-38.0)	2.89* (1.06-7.89)	1.93 (0.63-5.87)

**Husband involved in fights with other men**	No	737 (96%)	1		1	
	Yes	25 (4%)	6.05* (2.62-14.0)	5.16* (1.96-13.5)	2.1 (0.91-4.85)	-

^1^Adjusted for the variables Husband's type of work, Husband stole her money, Husband share his income, Husband has other woman, Husband drink alcohol, Husband witnessed his mother being hit as a child, Husband involved in fights with other men.

**^2^**Adjusted for the variables Women's economic independence, Geographical area, Husband's age, Husband's education, Husband share his income, Husband has other woman, Husband drinks alcohol, Husband witnessed his mother being hit as a child.

Sexual and physical violence had different risk profiles, although the common risk factor for the two types of violence was having an unfaithful husband. Sexual violence was associated with the husband being younger than 35 years (AOR 1.71; 1.19-2.47), and having less than nine years of education (AOR 2.07; CI 1.36-3.17). Women who reported sexual violence were more likely to be those having income independence (AOR 1.65; CI 1.08-2.32), and having an unfaithful husband (AOR 2.3; CI 1.09-4.88). Further analysis of woman's age (divided into two or three groups), education, parity or husband's type of work showed no association with women's reports of ever having experienced physical or sexual violence. Living in the highlands increased the odds for sexual violence, however after adjusting the variables of woman's income independency and husband's characteristics (age, education, share his income, has other woman, drinks alcohol and witnessed his mother being hit as a child), it was found to be no longer significant.

Only a small proportion of women reported that their husbands had a number of adverse psychosocial and behavioral characteristics (2-6%). Yet exposure to physical violence was strongly associated with these characteristics including having witnessed his mother being beaten by his father (AOR 5.16; CI 1.96-13.5), being unfaithful (AOR 8.15; CI 3.64-18.3), using alcohol (AOR 5.39; CI 2.46-11.8), and fighting with other men (AOR 5.16; CI 1.96-13.5).

Most women respondents (58-94%) supported at least four out of six statements that expressed traditional patriarchal gender norms (Table 2). Endorsement of these statements was most pronounced in the highlands among women married to husbands with less than 9 years of education and working in a non-agricultural setting (data not shown). Women who agreed that others should intervene in cases of violence had a lower risk of having been exposed to physical violence than those who disagreed. Women who agreed that a woman was obliged to have sex with her husband had a lower risk of having been exposed to physical violence than those who disagreed. Meanwhile, women's support for items of "good wife obeys her husband" and "a man should show who is the boss" were more likely to have experienced sexual violence than women who disagreed (Table 2).

**Table 2:** Association between women's attitudes towards marital norms, justifications for a man to hit his wife and woman's right to refuse sex and physical or sexual violence (OR, 95% CI).

ATTIDUDES	All women	Physical violence		Sexual violence	
	n = 765	OR	CI 95%	OR	CI 95%
**GENDER ROLES**					
**Good wife obeys husband**					
Disagree	42%	1		1	
Agree (traditional)	58%	0.71	0.45-1.12	1.44*	1.01-2.06
**Family problems should only be discussed with people in the family**					
Disagree	6%	1		1	
Agree (traditional)	94%	0.56	0.24-1.32	0.82	0.39-171
**A man should show who is the boss**					
Disagree	17%	1		1	
Agree (traditional)	83%	1.31	0.67-2.55	1.89*	1.09-3.26
**A wife is obliged to have sex with her husband**					
Disagree	19%	1		1	
Agree (traditional)	81%	0.48*	0.29-0.80	0.82	0.53-1.26
**Woman should be able to choose own friends**					
Agree	58%	1		1	
Disagree (traditional)	42%	1.32	0.83-2.08	0.99	0.69-1.41
**Others outside family should intervene**					
Agree	66%	1		1	
Disagree (traditional)	34%	0.58*	0.34-0.99	0.76	0.52-1.11
**JUSTIFICATION FOR HITTING**					
Hitting wife is never justified	42%	1		1	
Hitting wife is justified for one reason only	39%	1.47	0.88-2.45	1.19	0.79-1.764
Hitting wife is justified for two or more reasons	16%	1.69	0.89-3.20	2.09*	1.30-3.38
**JUSTIFICATION FOR REFUSING SEX**					
To refuse sex is justified for all four reasons	59%	1		1	
To refuse sex is justified for up to 3 reasons	31%	0.81	0.47-1.41	0.76	0.47-1.07
To refuse sex is justified for 1 reason or no reason at all	8%	2.98*	1.54-5.77	0.74	0.37-1.47

Most women endorsed none or only one of the reasons for a man to hit his wife, a finding that reveals a low common approval of violence. However, women who endorsed more justifications for male violence were more likely to have been exposed to both physical and sexual violence, although the association was only significant for sexual violence (OR 2.09; CI 1.30-3.38) (Table 2).

Slightly more than half of the women (59%) considered that refusing sex for all the stated reasons was justifiable. This view was most common among women with income independence (71%) (data not shown). Women who agreed to none or only one reason for refusing sex (8%) were more likely to be exposed to physical violence (OR 2.98; CI 1.54-5.77) but not to sexual violence compared with those who agreed to all four reasons (Table 2).

## Discussion

Sexual violence (22%) was reported more frequently than physical violence (11%) by women in our study [2]. We found that women who experienced sexual violence from an intimate partner were more likely to be those with income independence and married to a husband younger than 35 years who had less than 9 years of education. The attitudes and norms expressed by the women in our study confirm a high degree of gender inequality in married couples, particularly among women who were married to poorly educated husbands (less than 9 years). Exposure to physical violence was strongly associated with the husband's characteristics such as witnessing his mother being abused, being unfaithful, using alcohol, fighting with other men, or stealing the woman's money.

Previous studies of domestic violence have found a significant association between domestic violence and alcohol use [5]. However, the low rates of alcohol use in the study area, as a result of strictly enforced religious bans on drinking, might have contributed to the relatively low prevalence of physical violence. There are many faith-based schools *(madrasah) *in the hilly areas, and they have a strong social influence. On the other hand, gender norms that are based on culture and religion in this area confer absolute sexual autonomy of men over women. A study of religious participation and attitudes about intimate partner violence showed that conservative religious beliefs are risk factors for intimate partner violence [6]. The socioeconomic risk factors for sexual violence are similar to those described in other studies [7-11]. In our study, the relatively few husbands with adverse characteristics (2%-6%), as reported by their wives, were more likely to be both physically and sexually abusive. This is in line with other studies where male partners with a history of witnessing their father's abusive behavior and of having extramarital relationships had a significantly higher risk of being perpetrators themselves [5,11,12]. Men's lack of control over the household economy has also been found by other studies to be a risk factor for abuse [7,10].

Neither marital age nor educational discrepancy was associated with exposure to violence. This is in contrast to Ethiopian findings where educational discrepancy-women having higher education than their partners-was interpreted as a transitional process of challenging traditional values [13]. However, our study finds an increased risk of violence among women who have their own income. A study from rural Bangladesh also revealed similar findings, where affiliating with a village credit program for economic empowerment had precipitated domestic violence against women by their husbands [14]. However, studies from other settings have shown that having personal income also may become a protective factor against exposure to violence [1,8,9,11,15].

The high adherence to the norm that "family problems should only be discussed with people in the family" and the strong support for the statement that "others should intervene" in family conflicts indicate an internal conflict making it difficult for women to choose between seeking help and remaining silent for the sake of family harmony. A study among Asian women who live in the United States also illustrates this dilemma by showing the emphasis put on harmonious interpersonal relationships and interdependency in Asian families [16]. A study from an Arab Muslim setting describes marriage as a "conspiracy of silence" because the marital bond must be preserved at all costs [17].

There was a consistent pattern in the association between traditional attitudes to gender roles and violence in our study. A literature review on this specific topic [18] showed that women usually have less traditional attitudes towards sex role preference than men, but women who had experienced abuse lacked willingness to seek help due to their dominant role as the main breadwinner. This is reflecting a gender role conflict: guilty of performing as main provider and compensating for exhibiting masculine gender roles [18]. Women's endorsement of traditional norms concerning a husband's right to beat his wife has been shown to be associated with abuse [5,19], meanwhile 42% of the women did not think that a man had the right to beat his wife for any of the suggested reasons. Compared with other WHO multi-country study sites, our figure is low but still higher than the other southeast Asian sites [1]. However, 54% of the women considered beating was justified "if the wife is unfaithful". This is in line with the WHO study where more than 50% in eight out of 11 sites agreed to this statement [1,5]. Endorsement by 59% women to the statement that a woman has a right to refuse sex for all the stated reasons in the questionnaire is similar to the figures from two other southeast Asian sites in the WHO country study (Thailand and Bangladesh). They also had higher prevalence of sexual than physical violence [1]. The WHO finding that not wanting sex is not a reason for refusing sex was not strongly supported by our study because more than 90% considered a woman has the right to refuse sex "if she is sick", "if he is drunk", or "if he mistreats her".

The relatively strong support for women to refuse sex for all of the included reasons (59%) might indicate a greater "sexual autonomy" than in many other WHO settings [1]. However, our findings suggest that women who do not support (8%) the right of women to refuse sex were more likely to experience physical violence (OR 2.98; CI 1.54-9.77), while women who considered that hitting is justifiable for more than two reasons (16%) were more likely to experience sexual violence (OR 2.09; CI 1.30-3.38). Our finding also aligned with the WHO multi-country study where exposure to both sexual and physical violence was positively associated with greater approval of intimate partner violence [1]. Attitudes towards violence against women influence women's own responses to their victimization [20]. The more likely that the woman agrees with understanding of domestic violence, the more likely she is to blame herself for the assault, the less likely she is to report it to the police, and the more likely she is to experience long term negative effects [20]. In other words, those women exposed to their husbands' abuse perceived it as a random event in the relationship and thought there must be something they could correct in order not to be abused; but the abuse continued [21]. This phenomena reflects women in a state of "learned helplessness" due to the cycle of violence [22].

## Limitations of the study

The married women included in our study were recruited from a pregnancy cohort, which means that all of the respondents had experienced at least one pregnancy from their marriage, so they are not directly comparable with the women in the other WHO sites. However, the difference is not likely to have any significant influence on the results. The study relied on women's self-reported lifetime and 12-month experience (current) of physical and sexual violence. Because of the low current prevalence of physical violence (2%), the analysis was performed using lifetime prevalence. This may have introduced some recall bias: even though violent events are not something that a woman would actually forget easily.

Our final note, to avoid misunderstanding, translation from the original English questionnaire into Indonesia and back into English was done. But, since people in our study site speak Javanese, the field workers had translated some wordings into the local language for the respondents to understand the questions. This may have introduced some bias. However, through training and debriefing we have tried to minimize these problems.

## Conclusion

To conclude, our study confirms that Javanese women's experience of physical and sexual violence was associated with woman's income independence and husband's characteristics (younger than 35 years, and less than 9 years of education). There was a consistent pattern of association of woman's traditional attitude to gender role and woman's justification toward husband's abuse to physical and sexual violence. In general, women in our study lived in gender-based subordination within marriage relationships, maintained and reinforced by physical and sexual violence. Although the risk factors for sexual and physical violence were somewhat different, our findings suggest that community interventions designed to transform women's subordination norms into gender equality norms are needed to prevent gender based violence against women. Another community program that is specifically addressed towards men is also needed to reformulate ideas and promote non-violent masculinity values and norms.

## Competing interests

The authors declare that they have no competing interests.

## Authors' contributions

ENH participated in the design of the study, the data collection, was responsible for the preliminary analysis, and drafted the manuscript. UH contributed to the analysis and interpretation of the results and participated in the revision of the manuscript. MH designed the study protocol for the demographic aspects and was responsible for the arrangement of the field work in Purworejo. ME designed the study protocol for the domestic violence aspects, participated in the field work, and participated in the analysis and in the revision of the manuscript. MEm contributed to the analysis and interpretation of the results, and participated in the revision of the manuscript. All authors have seen and approved the final version of the manuscript.

## Authors' information

ENH is Head of the Masters Program, Faculty of Psychology, Ahmad Dahlan University, Yogyakarta, Indonesia. She is the former Executive Director of Rifka Annisa, a Women's Crisis Center in Yogyakarta, and still affiliated to Rifka Annisa to continues her advocacy work. She has a Masters degree in Public Health from Umea University, Sweden where she is now registered for a PhD. She has written several scientific publications on gender, women and gender based violence. Together with MH and ME, she has published a national report on domestic violence and women's health among Javanese women in Indonesia.

UH is Professor of Obstetrics and Gynecology at Uppsala University with an affiliation to the Department of Public Health and Clinical Sciences, Epidemiology and Global Health at Umeå University. He has published several papers on intimate partner violence in Ethiopia, Nicaragua and Sweden.

MH is Professor and Chairman, Department of Obstetrics & Gynecology, Faculty of Medicine, Gadjah Mada University, Yogyakarta, Indonesia. He obtained his PhD at the Centre for Clinical Epidemiology and Biostatistics, University of Newcastle, Newcastle, N.S.W., Australia. He has published more than 20 articles in national and international journals in the area of women's and maternal health. Together with ME and ENH, he published a national research report on domestic violence and women's health among Javanese women in Indonesia.

ME is vice president of research and programs at the International Center for Research on Women (ICRW). As vice president, she oversees ICRW's portfolios in economic development, gender, violence and rights, gender and HIV and gender, stigma and discrimination. She has more than 25 years of experience in international research and program work on gender inequity, domestic violence and sexual and reproductive health. ME is also a member of the Core Research Team of the World Health Organization (WHO) Multi-country Study on Domestic Violence and Women's Health. She has authored more than 20 books and articles in peer-reviewed journals on the prevalence and impact of gender-based violence on the health of women and children as well as ethical and methodological aspects of violence research.

MEm is Professor of Global Health at the Department of Clinical Sciences, Social Medicine and Global Health at Lund University. She has a special interest in the sociocultural aspects of health and has published several papers on intimate partner violence in Ethiopia and Tanzania.

## Pre-publication history

The pre-publication history for this paper can be accessed here:

http://www.biomedcentral.com/1472-6874/11/52/prepub
